# Disparities and interventions in the timeliness of endometrial cancer diagnosis and treatment in the United States: a scoping review protocol

**DOI:** 10.1186/s13643-021-01649-x

**Published:** 2021-04-13

**Authors:** Anna J. Najor, Dyda Dao, Jamie N. Bakkum-Gamez, Mark E. Sherman, Avonne E. Connor, Christopher C. Destephano

**Affiliations:** 1 Mayo Clinic College of Medicine and Science, Rochester, MN USA; 2 Department of Obstetrics and Gynecology, Mayo Clinic, Rochester, MN USA; 3 Department of Health Sciences Research, Jacksonville, FL USA; 4 Department of Epidemiology, Johns Hopkins Bloomberg School of Public Health, Baltimore, MD USA; 5 Department of Obstetrics and Gynecology, Mayo Clinic, Jacksonville, FL USA

## Abstract

**Background:**

Disparities in the stage at diagnosis of endometrial cancer (EC) account for a significant proportion of the disparities in morbidity and mortality experienced by vulnerable groups in the USA. Evidence suggests that disparities in timeliness of care and treatment play a significant role in stage at diagnosis. Despite an increase in literature on EC disparities, the issue remains largely unchanged. The objectives of this review will be to synthesize the evidence to identify important remaining research questions and inform future interventions to reduce the disparity in stage at diagnosis of EC in the USA.

**Methods:**

This scoping review protocol will use the five-step framework developed by Arksey and O’Malley. A literature search will be conducted from January 2000 onwards in PubMed, EMBASE, Scopus, and Cochrane CENTRAL databases. Studies on delays in care of EC will be included if they were published in English and reported findings for the US population. Two reviewers will independently screen all citations, full-text articles, and abstract data. The study methodological quality and bias will be appraised using appropriate tools. A narrative summary of findings will be conducted. Data analysis will involve quantitative (e.g., frequencies) and qualitative (e.g., content and thematic analysis) methods. The literature search, data extraction, and evidence synthesis will be informed by the Pathway to Treatment Model, which divides time to cancer care initiation into appraisal, help-seeking, diagnostic, and pre-treatment intervals. Results will be reported in accordance with the PRISMA statement.

**Discussion:**

EC disparities research is currently benefitting form a growing expectation that studies have a real impact on disparities. Patient, healthcare, and disease factors impact the amount of time patients spend in different intervals of the Pathway to Treatment Model, so research and interventions aimed at reducing disparities in EC survival should be designed with cognizance to how these factors impact their target population. Reviews on disparities in stage at diagnosis of EC exist but do not provide a comprehensive picture of the pathway to treatment. This review will seek to provide an expanded bedrock of evidence for future studies to build on as they aim to more actively reduce EC disparities.

**Trial registration:**

Open Science Framework (osf.io/v2zxy).

**Supplementary Information:**

The online version contains supplementary material available at 10.1186/s13643-021-01649-x.

## Background

Endometrial cancer (EC) is the fourth most common cancer diagnosed in women in the USA, and the incidence is increasing at such a rate that the roughly 50,000 cases detected in 2010 is expected to at least double by 2030 [1, 2]. There are significant disparities in the health outcomes of individuals with EC. Black individuals are more likely to be diagnosed at a later stage and more than twice as likely to die from EC than non-Hispanic White individuals [3]. High-risk histology is more prevalent in Black individuals [1, 4–6], but later stage at diagnosis also significantly contributes to this disparity [5]. Despite growing work on this topic, substantial gaps still exist in the evidence needed to inform interventions [6]. This scoping literature review will synthesize the evidence to identify important remaining research questions and inform interventions to reduce the disparity in stage at diagnosis.

Delays in diagnosis and treatment of EC likely contribute greatly to disparities in stage at diagnosis. A study of state-wide Surveillance, Epidemiology, and End Results (SEER) data found that African American individuals were still 41% more likely to present with advanced stage when controlling for age, tumor grade, and histology [7]. Another SEER study of 228,511 cases found that the disparity in survival is widened at later stages of diagnosis [8]. A recent literature review found a consistent 12–25% lower incidence of early-stage EC in African American individuals compared to White American individuals, significantly impacting the survival disparity [5]. Longer time to treatment accounts for a substantial amount of the disproportionate risk of presenting with advanced stage disease.

Other reviews have been published on disparities in EC outcomes, including the stage at diagnosis, that contribute to but do not complete a picture of the evidence pertaining to the factors that influence stage at diagnosis [6, 9–11]. One review studied the time between diagnosis and surgery, and the time between surgery and adjuvant treatment; however, the factors leading to disparities in stage at diagnosis were not analyzed [11]. Therefore, there is still a need to synthesize the evidence on the many factors with the potential to cause EC stage disparities across the entirety of patients’ journey from symptom onset to treatment.

Numerous factors may significantly contribute to delays in treatment and act on different time periods between first symptom occurrence till initiation of treatment. The Walter et al. Pathway to Treatment model, which elaborates on the Andersen Model of Total Patient Delay, provides a useful framework to identify sources of delay in cancer care [12, 13]. The Pathway to Treatment Model is divided into the following four time intervals:

- Appraisal interval: time from detection of bodily change(s) to perceiving reason to discuss symptoms with HCP
- Help-seeking interval: time from symptom onset to the first consultation with a care provider
- Diagnostic interval: time from the first consultation to diagnosis
- Pre-treatment interval: time from diagnosis to start of treatment

Additionally, the Pathway to Treatment Model divides factors that contribute to delays into three categories: patient factors (e.g., demographics, co-morbidities, social, cultural, previous experience), healthcare and systemic factors (e.g., access, healthcare policy, and delivery), and disease factors (e.g., site, size growth rate) [12].

A systematic scoping review will be conducted to examine factors that lead to disparities in EC stage at diagnosis in the USA, because unifying the current evidence on the pathway to treatment will facilitate designing targeted and effective research and interventions. Systematic scoping review methodology is particularly well suited for this aim given the wide spectrum of factors that the Walter et al. model shows may significantly contribute to later stage at diagnosis.

The National Institute on Minority Health and Health Disparities (NIMHD) Research Framework will be used to complement the Pathway to Treatment division of patient, healthcare and systemic, and disease factors [14]. This framework describes a matrix of interaction between *domains* of influence (biological, behavioral, physical/built environment, socio-cultural environment, health care system) and *levels* of influence (individual, interpersonal, community, and societal) on health outcomes of individuals, families/organizations, communities, and populations [14]. This conceptual framework supports the authors’ priority of creating knowledge that does not naturalize racial or other differences in health outcomes and allows for the representation of important nuance in the risk profiles within and between populations.

Given the significant social and political context regarding health disparities, the review context will be limited to the USA. The study population will include all of those with EC or who have the ability to develop EC. Current and historical discrimination from the systemic to individual level are considered for this review. The comparators will be the sub-population of a study that is identified as having no/less belonging to vulnerable population groups. The main outcomes of the study are defined as the disproportionate impact of variables in the Walter et al. model on vulnerable populations [12].

## Methods

The scoping review protocol has been registered within the Open Science Framework database (registration number: osf.io/v2zxy) and is being reported in accordance with the reporting guidance provided in the Preferred Reporting Items for Systematic Reviews and Meta-Analyses Protocols (PRISMA-P) statement [15, 16] (see checklist in Additional file 1). The proposed scoping review will be reported in accordance with the reporting guidance provided in the Preferred Reporting Items for Systematic Reviews and Meta-analyses (PRISMA) extension for Scoping Reviews (PRISMA-ScR) [17]. The scoping review methodology will be conducted in accordance with the framework proposed by Arksey and O’Malley and later by Levac et al. [18, 19]. The steps are outlined below and are as follows: (1) identifying the research question, (2) identifying relevant studies, (3) study selection, (4) data collection, and (5) reporting results.

### Eligibility criteria

The Population-Concept-Context (PCC) framework will be used to align the study selection with the research question [20]. To be included in the review, sources of evidence will need to include vulnerable population(s) with the ability to develop EC, address variable(s) outlined in the Walter et al. Pathway to Treatment Model [12] as they pertain to EC, and occur in the USA context.

We will exclude studies that have not undergone a peer-review process, were published before the year 2000, are literature reviews without meta-analysis, and are not published in the English language.

### Information sources and search strategy

The primary source of literature will be a structured search of electronic databases (from January 2000 onwards): PubMed, EMBASE, Scopus, and the Cochrane Central Register of Controlled Trials databases. The secondary source of potentially relevant material will be a search of Google Scholar. The references of included articles will be hand-searched to identify any additional evidence sources. The search strategy will be designed by a trained research librarian and peer reviewed by using the Peer Review of Electronic Search Strategies (PRESS) checklist [21]. A draft search strategy for PubMed is provided in Additional file 2.

### Screening and selection process

All articles identified from the literature search will be screened by two reviewers (A.J.N. and D.D.) independently. First, titles and abstracts of articles returned from initial searches will be screened based on the eligibility criteria outlined above. Second, full texts will be examined in detail and screened for eligibility. Third, references of all considered articles will be hand-searched to identify any relevant report missed in the search strategy. Any disagreements will be resolved by discussion, if necessary. A PRISMA flow diagram showing details of studies included and excluded at each stage of the study selection process will be provided [15]. Cohen’s kappa score will be calculated.

### Data charting process

The extraction of relevant data from the included studies, will be aided by the use of an organized charting process developed a priori [18]. The data extraction will be completed by two independent researchers. After data extraction of the first five articles, a third researcher (AJN) will review both copies of the data extraction to ensure consistency and quality. After duplicate data extraction is complete, one author (AJN) will again compare both copies for consistency and quality. If necessary, corresponding authors will be contacted via email to obtain additional data.

The data that will be extracted are organized according to the Walter et al. Pathway to Treatment Model [12]. Dependent variables are outcomes that pertain to Walter et al. Pathway to Treatment (knowledge of symptoms, help-seeking behaviors, etc.) [12]. Independent variables are tested for association with dependent variables and relate to health disparities. For the purpose of this review, this is defined as discrimination that results from prejudices including (but is not limited to) racism, homophobia, heteronormativity, ableism, capitalism, sexism, ageism, and the intersectional discrimination that results from combinations of these prejudices. This review considers the discrimination that targets populations through mechanisms including (but not limited to) poverty, limited transportation, under/unemployment, under/uninsured, limited educational opportunity, over-policing and incarceration. Since study population demographics are often reported in the literature rather than discriminatory practices and policies, vulnerable population status will also be used, since it serves as a rough proxy for exposure to discrimination. In the USA, vulnerable populations include (but are not limited to) Black, Indigenous, people of color, incarcerated, LGBTQ+, Muslim, low-income, undocumented, refugee, and disabled populations.

Study methods and design will also be extracted. This will include data source, location (geographic location of participant enrollment, whether single-center vs. multicenter), recruitment of participants (how participants were identified, response rate, enrollment rate, dropout rate), study population (eligibility criteria, exclusion criteria, how demographics were defined and measured), how the outcome was defined and measured. Disease characteristics will also be gathered, including age at diagnosis, race, ethnicity, socioeconomic status, year of diagnosis, cancer stage, performance status, histology, comorbidities, and disease risk factors. The supplementary Additional file 3 displays the proposed data extraction table.

### Quality assessment and evidence sources

Appropriate tools will be used to assess methodologic quality of studies. The Newcastle-Ottawa Scale criteria will be used for case control, cohort, and cross-sectional studies [22], the Critical Appraisal Skills Program checklist, for qualitative studies [23], the Cochrane Risk of Bias 2.0, for randomized trials [24], the Robins-I, for nonrandomized trials [25], and the AMSTAR-2, for systematic reviews [26]. Two reviewers will be involved in the quality assessment. Disagreements will be resolved through discussion between the two reviewers. If consensus cannot be reached, one of the senior authors will be consulted for a final decision.

Studies will be grouped by their pertinence to each of the Walter et al. variables [12] and risk of bias within each group will be assessed. If risk of bias is deemed high, synthesis will not occur. Consistency will be considered too low for synthesis if there are significant outcomes in different directions for a particular outcome. Precision will be considered too low for synthesis if confidence intervals are large for an outcome. Directness will be considered too low for synthesis if an intervention is only tested against a surrogate for a biologic outcome or there is no head-to-head comparison (for intervention comparisons) or if the individual studies are limited in their applicability to real populations. For example, if single-site studies pertaining to a particular outcome only report the impact of race on that outcome, then we will consider there to be inadequate information to know if the populations can be compared. Reporting bias will be considered too high for synthesis of the individual studies tend to publish specific outcomes. Publication bias and selective reporting will be assessed for meta-bias.

### Data synthesis

Data will be qualitatively synthesized if it meets the aforementioned quality criteria. Synthesis will occur separately for each of the Walter et al. variables. Even if outcomes meet criteria for synthesis, weaknesses regarding bias, consistency, precision, directness, reporting bias, dose-response association, plausible confounding that would change, the observed effect, and the strength of the association will be accounted for when performing qualitative synthesis. If the quality of the studies and results allow, we will also perform quantitative meta-analysis comparing vulnerable populations to non-vulnerable populations odds ratios or relative risks in eligible studies. For meta-analysis, a random effects model will be conducted and effect sized (e.g., odds ratio) with corresponding 95% confidence interval will be reported. When appropriate, the results will be analyzed using Stata version 16.0 [27].

Since observational research will be included, we will also assess dose-response association, plausible confounding that would change the observed effect, and strength of the association to determine whether synthesis is reasonable. Dose-response association will be taken into account but likely will not be utilized to disqualify synthesis since there can be variation in dose-response accounted for by other factors. Judging from the pilot literature search, there are likely many factors that can result in plausible confounding that would change an observed effect in the studies included in this study. For example, if study populations are not comparable, the results of the individual studies will not be synthesized. Observational studies will be assessed carefully for strength of association, since they are vulnerable to many confounding factors. If this appears to be prevalent for individual studies on a particular outcome, then the studies will not be synthesized.

The results will be reported narratively as well as in the data tables. Figures will include the PRISMA flow diagram [28]. The primary results tables will be organized by the four intervals in the Walter et al. intervals and sub-categorized by the disparities studied within each interval. A table summarizing study methodology and population, and a table reporting the quality appraisal of each study will be provided as supplements.

## Discussion

Since the evidence will be analyzed separately for each of the four time intervals in the theoretical model, analysis may be limited by a dearth of studies in an interval. When there are enough data for meaningful results, we will analyze outcomes by multiple demographic factors, for example, the impact of insurance status on surgical complications for Black people with female reproductive organs. If they occur, important protocol amendments will be submitted to the publishing journal as well as incorporated as updates to the protocol registered through Open Science Framework. We will utilize traditional end-of-study knowledge translation strategies, which include submission of study results to a national conference and publication in a peer-reviewed journal. In addition, we will seek out grass-roots patient advocacy groups to share our findings.

The field of EC disparities work and research is currently benefitting form a call to action to undermine racism rather than reify race as a biologic construct [6] and scholarship that raises the standard of good research to be that which is applied and has real impact on disparities [6, 29]. Patient, healthcare, and disease factors impact the amount of time patients spend in the appraisal, help-seeking, diagnostic, and pre-treatment intervals [12], so research and interventions aimed at reducing disparities in EC survival should be designed with cognizance to how these factors impact their target population. This review will seek to provide an expanded bedrock of evidence for future studies to build on as they answer the call to action.

## Supplementary Information

**Additional file 1.** PRISMA-P 2015 Checklist

**Additional file 2.** PubMed Search Strategy

**Additional file 3. **Proposed Data Extraction Tempalte

## Data Availability

The datasets used and analyzed during the current study are available from the corresponding author on reasonable request.

## Abbreviations

EC: Endometrial cancer
SEER: Surveillance, epidemiology, and end results
NIMHD: National institute on minority health and health disparities
