# Supplementary material for: Disparities and interventions in the timeliness of endometrial cancer diagnosis and treatment in the United States: a scoping review protocol
Source: Syst Rev. 2021 Apr 13;10:107. doi: 10.1186/s13643-021-01649-x (PMC8042979; doi:10.1186/s13643-021-01649-x)
Supplement: Supplementary file 2 — Additional file 2. PubMed Search Strategy [file 13643_2021_1649_MOESM2_ESM.docx]

The health disparity search term for PubMed was created by expanding the MEDLINE/PubMed Health Disparities and Minority Health Search Strategy (<https://www.nlm.nih.gov/services/queries/health_disparities_details.html>) and combined with a search term for endometrial cancer to yield the following search strategy:

((ethnic disparities[TIAB] OR ethnic disparity[TIAB] OR health disparities[TIAB] OR health disparity[TIAB] OR healthcare disparities[MH] OR health care disparities[TIAB] OR healthcare disparities[TIAB] OR health-care disparities[TIAB] OR health care disparity[TIAB] OR healthcare disparity[TIAB] OR health-care disparity[TIAB] OR healthcare disparities [MeSH Terms]) OR health status disparities[MH] OR (disparities in health[TIAB]) OR J Health Care Poor Underserved[Journal] OR J Health Dispar Res Pract[Journal] OR J Racial Ethn Health Disparities[Journal] OR (culturally competent care[MH] OR culturally competent care[TIAB] OR delivery of health care[MH:noexp] OR disparities[TIAB] OR health behavior[MH] OR health behavior[TIAB] OR health behaviors[TIAB] OR health inequality[TIAB] OR health inequalities[TIAB] OR health inequities[TIAB] OR health inequity[TIAB] OR health knowledge, attitudes, practice[MH] OR health related quality of life[TIAB] OR health-related quality of life[TIAB] OR health services accessibility[MH] OR health services, indigenous[MH] OR health services needs and demand[MH] OR health status disparities[MH] OR mass screening[MH] OR mass screening[TIAB] OR mass screenings[TIAB] OR patient acceptance of health care[MH] OR patient selection[MH] OR quality of health care[MeSH Major Topic:noexp] OR social class[MH] OR social class[TIAB] OR social determinants of health[MH] OR social determinants of health[TIAB] OR social disparities[TIAB] OR social disparity[TIAB] OR social factors[TIAB] OR social inequities[TIAB] OR social inequity[TIAB] OR socioeconomic factor[TIAB] OR socioeconomic factors[MH] OR socioeconomic factors[TIAB] OR socioeconomically disadvantaged[TIAB]) AND (African American[TIAB] OR African Americans[TIAB] OR African ancestry[TIAB] OR african continental ancestry group[MH] OR ageism[MH] OR AIAN[TIAB] OR Alaska Native[TIAB] OR Alaska Natives[TIAB] OR american native continental ancestry group[MH] OR apartheid[MH] OR Asian[TIAB] OR asian continental ancestry group[MH] OR Asians[TIAB] OR Black American[TIAB] OR Black Americans[TIAB] OR Caucasian[TIAB] OR Caucasians[TIAB] OR "Islam"[Mesh] OR Mohammedanism[tiab] OR Muslims[tiab] OR Muslim[tiab] OR Islamic Ethics[tiab] OR Ethic, Islamic[tiab] OR Ethics, Islamic[tiab] OR Islamic Ethic[tiab] OR disabled[TIAB] OR disabled persons[MH] OR disabled persons[TIAB] OR diverse population[TIAB] OR diverse populations[TIAB] OR emigrants and immigrants[MH] OR ethnic group[TIAB] OR ethnic groups[MH] OR ethnic groups[TIAB] OR ethnic inequalities[TIAB] OR ethnic population[TIAB] OR ethnic populations[TIAB] OR ghetto[TIAB] OR ghettos[TIAB] OR health services for persons with disabilities[MH] OR Hispanic[TIAB] OR hispanic americans[MH] OR Hispanics[TIAB] OR homeless[TIAB] OR homeless persons[MH] OR immigrant[TIAB] OR immigrants[TIAB] OR Indian[TIAB] OR Indians[TIAB] OR indians, north american[MH] OR inmate[TIAB] OR inmates[TIAB] OR jail[TIAB] OR jail population[TIAB] OR jail populations[TIAB] OR Latina[TIAB] OR Latinas[TIAB] OR Latino[TIAB] OR Latinos[TIAB] OR mexican americans[MH] OR medically underserved area[MH] OR medically uninsured[MH] OR minorities' health[TIAB] OR minority group[TIAB] OR minority groups[MH] OR minority groups[TIAB] OR minority health[MH] OR minority health[TIAB] OR minority population[TIAB] OR minority populations[TIAB] OR migrant worker[TIAB] OR migrant workers[TIAB] OR Native American[TIAB] OR Native Americans[TIAB] OR Native Hawaiian[TIAB] OR Native Hawaiians[TIAB] OR oceanic ancestry group[MH] OR Pacific Islander[TIAB] OR Pacific Islanders[TIAB] OR people of color[TIAB] OR poverty[MH] OR poverty[TIAB] OR poverty areas[MH] OR poverty area[TIAB] OR poverty areas[TIAB] OR prisoner[TIAB] OR prisoners[MH] OR prisoners[TIAB] OR race factors[MH] OR race factors[TIAB] OR race and ethnicity[TIAB] OR racial and ethnic minorities[TIAB] OR racial discrimination[TIAB] OR racial disparities[TIAB] OR racial disparity[TIAB] OR racial equality[TIAB] OR racial equity[TIAB] OR racial inequities[TIAB] OR racial inequity[TIAB] OR racial prejudice[TIAB] OR racial segregation[TIAB] OR racism[MH] OR refugees[MH] OR refugees[TIAB] OR rural health[MH] OR rural health[TIAB] OR rural health services[MH] OR rural population[MH] OR rural population[TIAB] OR rural populations[TIAB] OR sexism[MH] OR "Prejudice"[Mesh] OR Prejudices[tiab] OR Islamophobia[tiab] OR Islamophobias[tiab] OR Anti-Semitism[tiab] OR Anti Semitism[tiab] OR Anti-Semitisms[tiab] OR slum[TIAB] OR slums[TIAB] OR social discrimination[MH] OR social marginalization[MH] OR social segregation[MH] OR transients and migrants[MH] OR underserved[TIAB] OR undocumented immigrants[MH] OR medically uninsured[MH] OR uninsured[TIAB] OR urban health[MH] OR urban health services[MH] OR urban population[MH] OR urban population[TIAB] OR urban populations[TIAB] OR vulnerable population[TIAB] OR vulnerable populations[MH] OR vulnerable populations[TIAB] OR working poor[MH] OR working poor[TIAB] OR bisexuals[TIAB] OR bisexual[TIAB] OR bigender[TIAB] OR disorders of sex development[MH] OR disorders of sex development[TIAB] OR female homosexuality[TIAB] OR gay[TIAB] OR gays[TIAB] OR gender change[TIAB] OR gender confirmation[TIAB] OR gender disorder[TIAB] OR gender disorders[TIAB] OR gender dysphoria[TIAB] OR gender diverse[TIAB] OR gender-diverse[TIAB] OR gender diversity[TIAB] OR gender identity[MH] OR gender identity[TIAB] OR gender minorities[TIAB] OR gender non conforming[TIAB] OR gender non-conforming[TIAB] OR gender orientation[TIAB] OR genderqueer[TIAB] OR gender reassignment[TIAB] OR gender surgery[TIAB] OR GLBT[TIAB] OR GLBTQ[TIAB] OR health services for transgender persons[MH] OR homophile[TIAB] OR homophilia[TIAB] OR homosexual[TIAB] OR homosexuality[MH] OR homosexuality, female[MH] OR homosexuality, male[MH] OR homosexuals[TIAB] OR intersex[TIAB] OR lesbian[TIAB] OR lesbianism[TIAB] OR lesbians[TIAB] OR LGBBTQ[TIAB] OR LGBT[TIAB] OR LGBTI[TIAB] OR LGBTQ[TIAB] OR LGBTQI[TIAB] OR LGBTQIA[TIAB] OR nonheterosexual[TIAB] OR non-heterosexual[TIAB] OR non heterosexuals[TIAB] OR nonheterosexuals[TIAB] OR pansexual[TIAB] OR polysexual[TIAB] OR queer[All Fields] OR same sex [TIAB] OR sexual and gender disorders[MH] OR sexual and gender minorities[MH] OR sex change[TIAB] OR sex reassignment[TIAB] OR sex reassignment procedures[MH] OR sex reassignment surgery[MH] OR sex reassignment surgery[TIAB] OR sexual diversity[TIAB] OR sexual minorities[TIAB] OR sexual minority[TIAB] OR sexual orientation[TIAB] OR transgender*[TIAB] OR transgender persons[MH] OR transsexual*[TIAB] OR transman[TIAB] OR trans men[TIAB] OR transmen[TIAB] OR transsexualism[MH] OR transsexualism[TIAB] OR transwoman[TIAB] OR trans women[TIAB] OR transwomen[TIAB] OR two spirit[TIAB] OR two-spirit[TIAB] OR women who have sex with women[TIAB])) AND (endometrial neoplasms[mesh] OR endometrial neoplasm*[TIAB] OR endometrial cancer*[TIAB] OR endometrial carcinoma*[TIAB] OR endometrium cancer*[TIAB] OR Uterine Cancer*[TIAB] OR “Cancer of the Uterus”[TIAB] OR Uterine Neoplasm*[TIAB] OR Uterus Neoplasm*[TIAB]).
