# Supplementary material for: Disparities and interventions in the timeliness of endometrial cancer diagnosis and treatment in the United States: a scoping review protocol
Source: Syst Rev. 2021 Apr 13;10:107. doi: 10.1186/s13643-021-01649-x (PMC8042979; doi:10.1186/s13643-021-01649-x)
Supplement: Supplementary file 3 — Additional file 3. Proposed Data Extraction Tempalte [file 13643_2021_1649_MOESM3_ESM.docx]

**Data Extraction Template**

- Citation
- Study characteristics
  - Design
  - Location
  - Setting
  - Time interval
- Participants
  - Inclusion and exclusion criteria
  - Number of participants
  - Response rate
- Results
  - Relevant outcome (to Pathway to Treatment)
  - Variables tested for association with relevant outcome
  - Descriptive statistics of demographic variables
  - Analysis
  - Findings
